# Development of Human Rhinovirus RNA Reference Material Using Digital PCR

**DOI:** 10.3390/genes14122210

**Published:** 2023-12-14

**Authors:** Dong U Ju, Dongju Park, Il-Hwan Kim, Seil Kim, Hee Min Yoo

**Affiliations:** 1 Biometrology Group, Korea Research Institute of Standards and Science (KRISS), Daejeon 34113, Republic of Korea; 2 School of Biomedical Engineering, Korea University, Seoul 02841, Republic of Korea; 3 Department of Precision Measurement, University of Science & Technology (UST), Daejeon 34113, Republic of Korea

**Keywords:** reference materials (RMs), human rhinovirus (RV), reverse transcription-quantitative polymerase chain reaction (RT-qPCR), droplet digital PCR (ddPCR)

## Abstract

The human rhinovirus (RV) is a positive-stranded RNA virus that causes respiratory tract diseases affecting both the upper and lower halves of the respiratory system. RV enhances its replication by concentrating RNA synthesis within a modified host membrane in an intracellular compartment. RV infections often occur alongside infections caused by other respiratory viruses, and the RV virus may remain asymptomatic for extended periods. Alongside qualitative detection, it is essential to accurately quantify RV RNA from clinical samples to explore the relationships between RV viral load, infections caused by the virus, and the resulting symptoms observed in patients. A reference material (RM) is required for quality evaluation, the performance evaluation of molecular diagnostic products, and evaluation of antiviral agents in the laboratory. The preparation process for the RM involves creating an RV RNA mixture by combining RV viral RNA with RNA storage solution and matrix. The resulting RV RNA mixture is scaled up to a volume of 25 mL, then dispensed at 100 µL per vial and stored at −80 °C. The process of measuring the stability and homogeneity of RV RMs was conducted by employing reverse transcription droplet digital polymerase chain reaction (RT-ddPCR). Digital PCR is useful for the analysis of standards and can help to improve measurement compatibility: it represents the equivalence of a series of outcomes for reference materials and samples being analyzed when a few measurement procedures are employed, enabling objective comparisons between quantitative findings obtained through various experiments. The number of copies value represents a measured result of approximately 1.6 × 10^5^ copies/μL. The RM has about an 11% bottle-to-bottle homogeneity and shows stable results for 1 week at temperatures of 4 °C and −20 °C and for 12 months at a temperature of −80 °C. The developed RM can enhance the dependability of RV molecular tests by providing a precise reference value for the absolute copy number of a viral target gene. Additionally, it can serve as a reference for diverse studies.

## 1. Introduction

The human respiratory system contains several viral families, including Orthomyxoviridae, Pneumoviridae, Picornaviridae, Coronaviridae, and others [1,2,3]. Additionally, individuals with asthma are vulnerable to respiratory viruses, including rhinoviruses (RV), respiratory syncytial virus (RSV), influenza virus, parainfluenza virus, adenovirus, and coronavirus [4,5,6,7]. RV are the most frequent viruses among the major causes of asthma exacerbation, contributing to approximately 80% of asthma exacerbations in children and adults during viral infections [8,9,10,11,12].

RV belong to the positive-sense RNA viruses of the Picornaviridae family and are responsible for respiratory infections that occur worldwide throughout the year [13,14,15]. RV infections can lead to various respiratory illnesses, causing significant morbidity across all age groups. Common diseases include those caused by infections in the upper respiratory tract, such as tympanitis and sinusitis, while infections in the lower tract can exacerbate conditions like bronchitis, pneumonia, asthma, and cystic fibrosis in children [16,17,18]. Severe cases and fatalities due to RV are more prevalent in vulnerable populations like the elderly and immunocompromised infants [19,20,21]. RV infections are often detected alongside other respiratory viruses, and they may not manifest symptoms for an extended period [22]. Previous studies have shown that human rhinoviruses (RV) induce an interferon (IFN) response in differentiated respiratory epithelial cells that confers protection against subsequent Influenza A virus (IAV) infections [1,23]. RV have a high prevalence in the human and have been singled out for their ability to negatively interact at the host level with IAV, resulting in a potent IFN response, and for their sensitivity to the IFN response of severe acute respiratory syndrome coronavirus 2 (SARS-CoV-2) [24,25,26]. The worldwide spread of SARS-CoV-2 has resulted in a pandemic, causing patients worldwide to suffer from coronavirus disease (COVID-19). The symptoms of COVID-19 range from mild to severe pneumonia. In particular, the clinical features of RV infections can resemble those of COVID-19 [27,28,29].

While there is an evident medical necessity, there is currently no clinically available drug directly addressing RV infection [30]. Developing a universal anti-RV drug or vaccine has proven challenging due to various factors, including the substantial and continually increasing number of RV strains exhibiting a relatively uniform geographic distribution and high levels of sequence variability between strains [15,31]. Given that RV have positive-sense single-stranded RNA as their genome [32,33], they could serve as an optimal target for DNA enzymes in an innovative antisense-based treatment approach [30,34]. DNA enzymes specifically bind to RNA target molecules and subsequently degrade them through enzymatic cleavage, presenting a promising avenue for addressing RV infections [35,36]. Furthermore, several studies have tested the ability of siRNA molecules to induce the inhibition of RV replication in cell culture experiments [37]. The results revealed that many siRNA molecules are effective in triggering RNA silencing, leading to the effective suppression of viral replication [38]. Therefore, it demonstrates that siRNA molecules derived from the RV genome robustly inhibit RV replication within cells [38].

Research exploring the influence of RV viral load on infection severity, symptoms, and outcomes necessitates clinical samples containing RV RNA that is precisely and comprehensively quantified, both qualitatively and quantitatively. Quantitative measurement of RV in patients who test positive for RV despite showing no symptoms is crucial [39,40]. Moreover, high-precision RV viral load assessments are essential for evaluating the effectiveness of potential antiviral drugs targeting RV [41]. According to findings involving viruses that cause other respiratory diseases, the viral load shares a correlation with disease severity; for this reason, it is vital to conduct RV viral-load measurements with patients who test positive for RV despite showing no symptoms [39,40,42,43]. Furthermore, the effectiveness of potential antiviral drugs that target RV requires RV viral-load assessments of high precision in their evaluation [41].

The importance of accurate qualitative and quantitative analysis of RV in the evaluation of antiviral drug efficacy cannot be understated. Although the determination of RV infection can be sufficiently performed through reverse transcription-quantitative polymerase chain reaction (RT-qPCR) to qualitatively detect RV, RV RNA needs to be precisely quantified in practical clinical samples when studying the correlations between RV virus transmission and patient symptoms and outcomes. Although viral copy quantification in a sample is possible through the combination of RT-qPCR, such a method may face challenges due to primer and probe sequences having bases that do not match with those of certain viral sequences, resulting in amplification issues and potentially inaccurate results [44,45,46,47].

qPCR has several limitations. It is highly sensitive, rendering it susceptible to contamination, and even a small amount of contaminating DNA or RNA can result in false-positive results [48,49]. Accurate quantification in qPCR often relies on the availability of suitable reference standards, and the choice and quality of these standards can impact the reliability of the results [50,51]. There are limits to the dynamic range and sensitivity of qPCR, making extremely low or high target concentrations challenging to accurately quantify [52]. Inhibitory substances in the sample, such as contaminants or substances from the sample matrix, can interfere with the PCR reaction, affecting the accuracy of the results [53,54,55]. In comparison to RT-qPCR, reverse transcription droplet digital PCR (RT-ddPCR) offers a higher quantification accuracy due to its reduced susceptibility to factors like standard curve variations, PCR efficiency, and primer–probe mismatches [56,57,58]. For these reasons, RT-qPCR is being replaced by or complemented with RT-ddPCR in an increasing number of studies and applications [59,60,61,62,63,64]. RT-ddPCR allows for the quantification of sequence-specific RNA using pre-selected gene copy number concentrations, eliminating the need for further calibration. Therefore, the utilization of RT-ddPCR may yield superior outcomes when quantifying RV RNA [65,66,67,68].

The application of quantitative methods for DNA/RNA analysis presents greater challenges than some researchers may anticipate. Failure to adhere to rigorous practices can result in inaccurate quantification, directly impacting the reproducibility of published data [69,70]. Enhancing data comparability and reproducibility necessitates a comprehensive description of experimental results for qPCR or ddPCR, as emphasized in the MIQE and digital MIQE guidelines [71,72]. A meticulous evaluation of these quantification protocols, encompassing a substantial number of samples and assays, is also imperative for assessing technical optimizations and limitations [73].

RMs are fundamental to viral diagnostics, particularly in methods including PCR, qPCR, and ddPCR. They provide a standardized benchmark for assay development and validation, ensuring consistency and reliability across diverse laboratories and experiments [74,75]. RMs permit instrument calibration and enable the establishment of a dependable quantitative scale [76]. Furthermore, these materials are used to maintain the quality of diagnostic assays by monitoring performance in each run and detecting any deviations from expected results, ensuring the reliability of the diagnostic process [77,78]. Moreover, correlating RMs with clinical outcomes enhances the comprehension of the clinical relevance of diagnostic assays [79,80]. In summary, RMs play a crucial role in guaranteeing the accuracy, dependability, and comparability of viral diagnostic assays. Consequently, they contribute to the standardization, quality control, and overall advancement of diagnostic approaches in virology.

RT-qPCR and RT-ddPCR are methods used for evaluating the qualitative and quantitative features of the RV RM. The RM holds a pivotal role in guaranteeing the accuracy of measurement materials [81,82]. With molecular diagnostics being prevalent globally to diagnose infectious diseases, it is necessary to comprehensively assess the RM, a stable and uniform substance with distinct traits. The RV RM ought to exhibit reliable and objective metrics, allowing for the efficient evaluation and comparison of varied diagnostic kits [68,83,84]. Appropriate quantification procedures were consistently applied to demonstrate accuracy. The conformity and resilience of the RV RM were verified in this investigation, in adherence with regulations defined in ISO Guide 17034 [85].

## 2. Materials and Methods

### 2.1. Cell Cultures and Preparation of RNA

For human cell lines, MRC-5 cells (ATCC CCL-171) from the American Type Culture Collection (ATCC) and rhinovirus (NCCP40602) from the National Culture Collection for Pathogens (NCCP) were used. The culture media were MEM/EBSS with L-Glutamine (Cytiva HyClone^TM^, Seoul, Korea) and Earle`s Balanced Salts (0.1 μm sterile filtered, Cytiva, Seoul, Korea) supplemented with heat-inactivated and filtered fetal bovine serum (FBS), 1% Non-Essential Amino Acids (100×, Gibco, Waltham, MA, USA), 1% Penicillin-streptomycin (10,000 U/mL, Gibco, Waltham, MA, USA), and 1% Sodium Pyruvate (100 mM, Gibco, Waltham, MA, USA). The MRC-5 cells were maintained in culture media with 10% FBS and cultured in a 5% CO_2_ incubator for 2 weeks under a temperature of 37 °C after thawing. The cells (Passage 14) were inoculated with rhinovirus in culture media with 2% FBS. The virus was cultured at 34 °C in a 5% CO_2_ incubator for 4 days in a Biosafety Level 2 (BSL-2) laboratory. The extraction of viral genomic RNA was performed using the QIAamp Viral RNA Mini kit (Qiagen, Hilden, Germany) by following the manufacturer’s guidelines. The extracted RNA was measured using QuantiFlour^®^ RNA System and Quantus (Promega, Madison, WI, USA) to check the concentration. The estimated RNA was stored at −80 °C until use. The concentration of the RNA copy number was measured by one-step RT-ddPCR and one-step RT-qPCR methods using assays developed in-house (Table S1). The reporter and quencher for the probe are 5′-HEX (or FAM) and 3′-BHQ1, respectively. The DiaPlexQ™ (Solgent, Daejeon, Korea) commercial RV16 kits were used, and RT-ddPCR was conducted following the manufacturer’s instructions.

### 2.2. Reverse Transcription Droplet Digital PCR (RT-ddPCR)

This experiment was conducted with reference to the primer–probe concentrations used in a previous experiment [68,86]. The prior experiment utilized identical equipment, and since RV share similarities with SARS-CoV-2 in respiratory virus infection, we determined the primer–probe mix concentration by following the protocol of the previous experiment. The RT-ddPCR experiment used a supermix for the probes (Bio-Rad Laboratories, Hercules, CA, USA) with the QX200 system (Bio-Rad Laboratories, Hercules, CA, USA). The total volume of the reaction mixture was 20 µL (5 μL of supermix, 2 μL of reverse transcriptase, 1 μL of 300 mM dithiothreitol (DTT) from a One-Step RT-ddPCR Advanced Kit for Probes (Bio-Rad Laboratories, Hercules, CA, USA), along with 5 μL of template, 4 μL of nuclease-free water (Invitrogen, Waltham, MA, USA), and 1 μL of 10 μM forward primer, 1 μL of 10 μM reverse primer, and 1 μL of 5 μM probe labeled with FAM), and the manufacturer’s instructions were referenced for the preparation process. The RT-ddPCR process started with a 60 min reverse transcription step at 42 °C followed by a 10 min enzyme activation step at 95 °C. This was followed by 70 cycles with a 20% ramp rate of denaturation at 95 °C for 30 s and 150 s of annealing and extension at 60 °C, ending with a 10 min enzyme deactivation step at 98 °C.

### 2.3. RT-qPCR Analysis

The RT-qPCR analysis was performed using the StepOne and StepOnePlus Real-Time PCR systems from Thermo Fisher Scientific, USA, along with the One Step PrimeScript RT-PCR Kit (Perfect Real Time) supplied by Takara (Takara Bio Inc., Kusatsu, Japan). The total reaction volume for the RT-qPCR was 20 μL, and the reaction mixture was carefully prepared according to the manufacturer’s detailed instructions. The DiaPlexQ™ (Solgent, Daejeon, Korea) commercial RV16 kits were used, and RT-qPCR was conducted following the manufacturer’s instructions.

### 2.4. Homogeneity and Stability Tests

Ten RV RM-positive tubes were randomly selected for RT-ddPCR measurements using gene-specific assays to assess the between-bottle homogeneity. The homogeneity between the bottles was determined by calculating the difference between the method repeatability and the relative standard deviation (RSD) observed among the bottles for each gene target. The reproducibility of the method was calculated by determining the RSD of the repeated measurements on the same specimen in one trial. Furthermore, short-term shipping stability, long-term stability, and freeze–thaw durability were assessed with up to three positive tubes for each experiment with triplicate repetitions. For the short-term stability test, three randomly selected sets of RMs stored at −70 °C were placed under 4 °C and −20 °C. Copy numbers were measured in samples stored for 0, 4, and 7 days. In the long-term stability test, we randomly selected and thawed one or three samples stored at −70 °C and measured the number of copies after 1, 3, 6, and 12 months in storage. Comparative analysis was conducted between the produced results and the homogeneity results. Three sets of RMs stored at −80 °C were subjected to three cycles of thawing at 4 °C and freezing again at −80 °C in the freeze–thaw test. All these tests provided useful information regarding the stability and reliability of the RV RM.

### 2.5. Unvertainty and Statistical Analyses

Each of the sources of uncertainty considered was assessed on an individual basis by carrying out Type A and Type B assessments separately for each target gene. Standard deviations were computed from independent experiments. The relative standard deviation (RSD) of the manual thresholds was calculated as the RSD of three different thresholds of over ten independent measurements. Furthermore, the standard uncertainty from partition volume variability was computed under the assumption of a uniform rectangular distribution over the range of reported drop volumes [62,87,88]. Type A and B RSDs were combined by taking the positive square root of the summed squared RSDs to produce a combined relative standard uncertainty. Combined standard uncertainties for each target were combined to produce expanded uncertainties with a coverage factor of k = 2.2 (95% confidence level, degrees of liberty = 11). Experiments were analyzed by Welch’s t-test (two-tailed) using Microsoft Excel 2016 (Microsoft, Redmond, WA, USA) and were repeated at least in triplicate or otherwise as indicated in the corresponding figure. The mean ± standard deviation is indicated by error bars in the graphical data. When the *p*-value was less than 0.05, statistical significance was assumed.

## 3. Results

### 3.1. RV Reference Material Design and Preparation Processes

**Figure 1:**
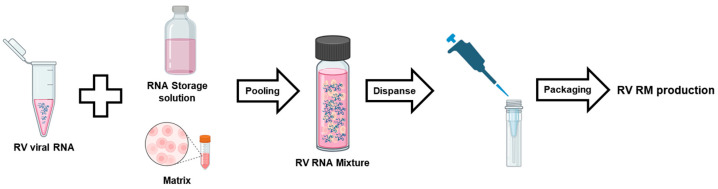
A schematic overview of the procedures RM production.

Figure 1 summarizes the overall scheme to produce the KRISS 111-10-536 RM (batch 1). First, rhinovirus is infected into host cells to initiate the culture. Next, viral genomic RNA is extracted using a kit following the provided guidelines, and the concentration of the extracted RNA is measured and analyzed. Following the concentration measurement, RT-ddPCR is employed to determine the virus genome copy number, after which the samples are diluted to achieve an approximate copy number of 10^5^ copies/µL or higher. Using the prepared samples, the production of over 300 vials of RV RM is carried out. Each vial contains 100 µL of positive materials and is immediately stored at −80 °C.

### 3.2. RV RMs Serial Dilution

**Figure 2:**
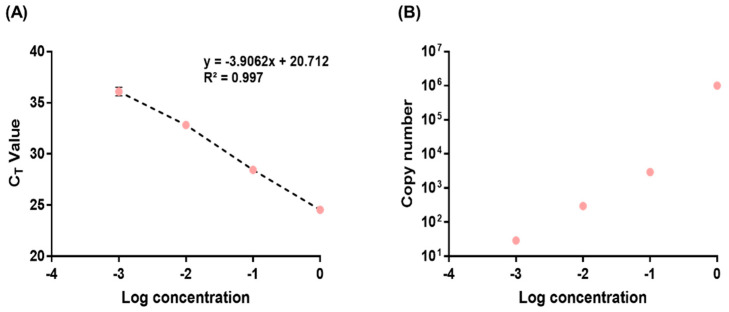
Human RV RM serial dilution. Detection of human RV based on assays by qPCR and ddPCR. (**A**) Ct value (qPCR) and (**B**) copy number (ddPCR) of rhinovirus using the assay. All experiments were conducted three times and the data presented represent the average values obtained.

Due to the involvement of a matrix in the RM production process, obtaining an accurate OD value for RM RNA is challenging. For this reason, qPCR was employed to identify a suitable Ct value (Figure 2A), approximately ranging from 28 to 32, indicative of good ddPCR results. Subsequently, ddPCR was carried out using the dilution concentration determined from the qPCR results (Figure 2B and Figure S2). After identifying a dilution concentration exceeding the initially estimated copies/μL of 1 × 10^5^, the experiment was conducted. The dilution factor used in this experiment was 10^−2^.

In this study, we conducted a comparative experiment between the reference material (RM) and the RV16 template, a commercial kit template, using the assay (Figure S1). The results revealed detection not only with the RM but also with the commercial kit template employing the assay used in this study. Therefore, it highlights the applicability of the assay not only for the RM but also for templates from commercial kits.

### 3.3. RV RMs Homogeneity Test

**Figure 3:**
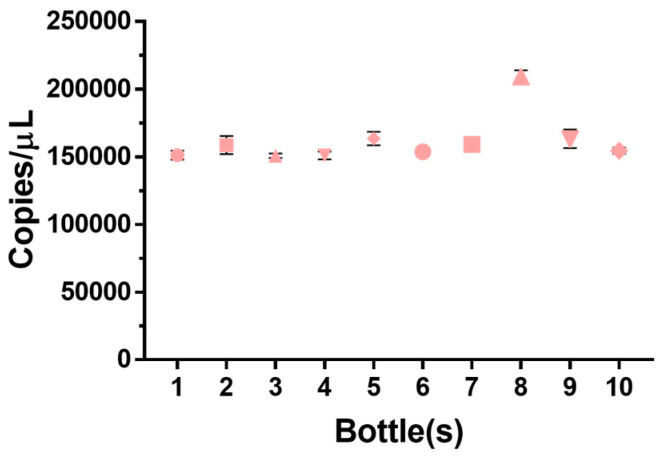
RMs homogeneity test. A homogeneity experiment was performed by randomly selecting 10 of the produced RMs. Error bars represent the standard deviation at each data point, calculated based on the mean of the replicated measurements (*n* = 3). Homogeneity values for the gene among bottles are presented as percentages. As a result, the average value is 1.6 × 10^5^ copies/mL, and the calculation results of the relative standard deviation (RSD) of about 11% and the standard uncertainty of 3.2% are shown. Therefore, it was confirmed that the produced RM was made homogeneously.

A total of 300 RV RM samples were selected randomly, and 10 samples were used for a homogeneity assessment as per ISO 17034, which mandates that at least 10 units in a reference material batch must be evaluated (Figure 3). This experiment assessed the homogeneity of the RM through RT-ddPCR. As a result, the average value was 1.6 × 10^5^/µL. The calculation results show a relative standard deviation (RSD) of 10% and a relative standard uncertainty of 3.2% (Table 1). The observed variation in the homogeneity test could be due to several factors, including the inherent instability of RNA’s structure, potential errors during the experimental process, and discrepancies that may arise while aliquoting RNA into vials. However, the study’s results revealed that the copy numbers fell within an acceptable error range, indicating that the KRISS RM batch shows homogeneity. Therefore, the homogeneity test provided valuable insights into the RM preparation, reducing the risk of significant errors in specific samples, despite these factors.

### 3.4. Short- and Long-Term Stability of RV RMs

**Figure 4:**
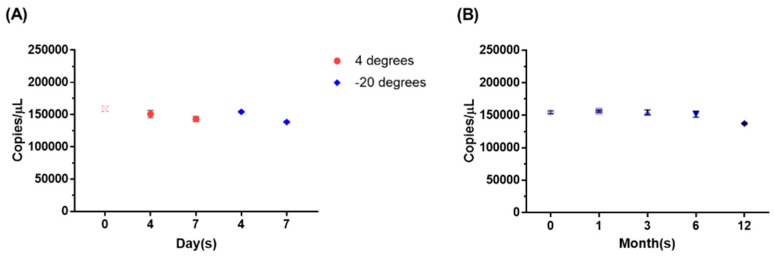
Short-term and long-term stability of RV RMs. (**A**) The short-term stability of the RM was confirmed after 4 days and 7 days at 4 °C and −20 °C. As a result, the results showed an almost negligible difference at both 4 °C and −20 °C. (**B**) Long-term stability after 1, 3, 6, and 12 months was confirmed. RM was stored at −80 °C.

The reference materials were subjected to both short-term and long-term stability studies to determine the stability characteristics over typical transport periods. The standard materials were stored at −20 °C and 4 °C for 0, 4, and 7 days, and the copy numbers of viral RNAs were measured. When stored at these temperatures, both showed no significant impact on the copy number values for up to 4 days. However, after 7 days of storage, there was a slight decrease in the copy number values, although they remained within the range of uncertainty, indicating relatively stable storage conditions (Figure 4A and Figure S3A).

To evaluate long-term stability, the copy number of viral RNAs in the substance was measured after being stored under specific conditions at −80 °C for 1, 3, 6, and 12 months. The results from storage at −80 °C for 1 month, 3 months, and 6 months showed no significant changes in the copy number values. However, the 12-month results indicated a tendency of decreased copy number values, but the extent of the decrease remained within the range of uncertainty, confirming that the substance remained relatively stable up to 12 months (Figure 4B).

Therefore, for more stable storage and reliable results, it is recommended to store the reference materials at −80 °C and use them within 7 days after storage at 4 °C. Additionally, using the materials within 12 months will likely yield more stable and reliable results.

### 3.5. RV RM Freeze–Thaw Repeated Test

**Figure 5:**
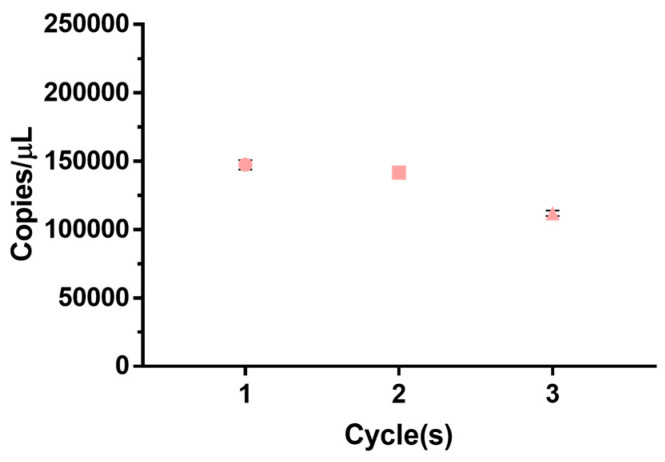
RV RM freeze–thawing repeated test. An experiment was conducted to confirm the change in the number of copies when the freezing and thawing of the RM were repeated. The experiment was repeated in the order of thawing at 4 °C and freezing at −80 °C. As a result, it shows the result that the change in the number of copies appears insignificant until the third repetition. Therefore, it shows results that can be used stably even in repeated freeze and thawing.

In addition, stability assessments were performed during freeze–thaw cycles to account for the instability of viral particles and RNA during these processes. The experiment included a total of three cycles in which the samples were thawed at 4 °C and then frozen at −80 °C. The results showed that there were minimal changes in copy numbers up to the third cycle (Figure 5 and Figure S3B). However, these changes were within the range of copy number errors, indicating that they had a negligible effect. Therefore, the results suggest that the reference materials can be used reliably even during repeated freeze–thaw cycles.

## 4. Discussion

The rhinovirus reference material (RM) is derived from viral RNA and has an approximate copy number concentration of 1.6 × 10^5^ copies/µL, which exceeds the threshold of ~10^5^ copies/µL. These RM values are used as specific reference points in various molecular testing applications, including RT-qPCR, next-generation sequencing [89], and CRISPR nuclease-based detection [90,91]. The broad applicability of the KRISS 111-10-536 RM enhances the reliability of molecular testing, and a robust standard for the comparison of different methods based on RT-qPCR is presented in the form of reference values in copy number units, enabling comparisons based on Cq values.

The primary source of measurement uncertainty for the developed reference material arises from the pre-analytical processes, particularly RNA extraction and RNA handling. The combined uncertainty values were consolidated and are presented in Table 1. These values were derived from RNA extracted from a subset of the RM employing a dedicated commercial viral RNA extraction kit. It was demonstrated that the efficiency of RNA extraction significantly relies on both the chosen method and the skills of the operator [92,93].

The validated RV RMs have demonstrated high homogeneity and stability between vials, providing reliable and consistent results. These developed RMs serve as accurate reference values for the absolute copy number of the viral target gene, thereby enhancing the reliability of RV molecular assays. In addition, they can be used as reference standards in various research studies. Unlike qualitative standards such as positive controls, the KRISS 111-10-536 RV RM provides reference values in terms of copy number concentration of the target RNA. In summary, the KRISS 111-10-536 RV RM plays a critical role in establishing measurement standards for RV molecular testing, contributing to improved accuracy and reliability in the field.

Human rhinoviruses are currently classified into three species within the Enterovirus genus of the Picornaviridae family: RV-A, RV-B, and RV-C [94,95,96,97,98]. However, the RV RM used in this study is defined under the broader category of rhinoviruses. While it can detect symptoms associated with all RV strains, it may not specifically distinguish individual species within the group. Furthermore, there is no existing research on the association between RV and other respiratory diseases in this study. Therefore, the relationship and interactions between RV and other respiratory diseases should be studied further using the newly developed KRISS 111-10-536 RV RM.

Physical examinations and a review of a patient’s medical history are generally necessary steps when diagnosing rhinovirus infections [15,99,100]. However, in patients with severe symptoms and complications, the diagnosis process may require a differentiated diagnosis, as similar symptoms and complications can be caused by other common viruses, such as coronaviruses, parainfluenza viruses, and adenoviruses [101,102,103]. Rhinovirus infections can be diagnosed through antigen detection and nucleic acid detection methods. RT-qPCR and RT-ddPCR have been shown to be methods [68,86] that are significantly more sensitive in terms of detecting these viruses than cell cultures. Antigen tests enable point-of-care testing (POCT), which comes with the advantage of being able to produce results within minutes without having to rely on specialized laboratory equipment or highly trained personnel [104,105]. Despite the relatively low sensitivity of antigen tests when compared to virus isolation methods and nucleic acid detection methods, such tests offer advantages in terms of convenience, accessibility, and cost-effectiveness [106,107]. The developed KRISS 111-10-536 RV RM in this study can be utilized as a material for the development of an easy PCR-based diagnostic test kit (Figure S1), indicating the potential for a rapid response during future outbreaks of respiratory-related pandemics.

## 5. Conclusions

Human rhinovirus (RV) reference material (RM) plays a crucial role as a specific reference for various applications involving molecular testing, such as RT-ddPCR and next-generation sequencing, providing reliable and accurate results. Validated RV RMs exhibit high homogeneity and stability, serving as valuable reference standards for absolute viral gene copy numbers, thereby enhancing the reliability of RV molecular tests and research studies. Further investigation is needed to explore the relationship between RV and other respiratory diseases using the newly developed KRISS 111-10-536 RV RM. Additionally, this RM can facilitate the development of user-friendly PCR-based diagnostic test kits.

## Figures and Tables

**Table 1:** Reference values of the KRISS 111-10-536 RV RM batch 1.

Homogeneity	Value
Average	1.6 × 10^5^ copies/μL
Standard deviation	1.7 × 10^4^ copies/μL
Relative standard deviation	10.76%
Relative standard uncertainty	3.2%
Expanded uncertainty	4.5 × 10^4^ copies/μL
*k* (95% level of confidence)	2.1

## Data Availability

The data presented in this study are available in the article.

## Supplementary Materials

The following supporting information can be downloaded at: https://www.mdpi.com/article/10.3390/genes14122210/s1, Figure S1: Comparison of human RV RM vs. commercial kit templates. Experimental comparison between human RV RM and commercial control samples using the RV assay for (A) qPCR and (B) ddPCR; Figure S2: 1D-Amplitude plots of a ddPCR using human RV RM; Figure S3. RT-qPCR data for the RM. We conducted qPCR experiments for the previously performed (A) Short term stability and (B) freeze and thawing. The results indicate a similarity to our previous ddPCR data; Table S1: dPCR oligonucleotide design and target information; Table S2: dPCR protocol; Table S3: dPCR assay validation and data analysis [108,109,110,111,112,113,114].
