# Supplementary material for: Development of Human Rhinovirus RNA Reference Material Using Digital PCR
Source: Genes (Basel). 2023 Dec 14;14(12):2210. doi: 10.3390/genes14122210 (PMC10742479; doi:10.3390/genes14122210)
Supplement: Supplementary file 1 [file genes-14-02210-s001.zip › genes-2671708-supplementary.pdf]

Supplementary Materials

1. RNA Templates

The information regarding RNA templates for RV is detailed in main Section 2.1 of the Materials and Methods, outlining the composition and conditions of the cell culture.

2. dPCR oligonucleotide design and target information

The RV genome spans approximately 7.2 kb, beginning with a 5' non-coding region (5'NCR). Subsequent to this, there exists a lengthy open reading frame tasked with encoding four structural icosahedral capsid proteins (VP4, VP2, VP3, and VP1). Additionally, the genome codes for seven non-structural proteins—2A, 2B, 2C, 3A, 3B, 3C, and 3D—before concluding with a brief 3' untranslated region (3'UTR) and a poly A tract [1]. Genetically and antigenically, Human Rhinoviruses (HRVs) exhibit significant diversity [2,3]. With over 140 serotypes identified, they are categorized into three species: HRV species A (HRV-A; consisting of 74 serotypes), HRV-B (with 25 serotypes), and a distinct third genotype known as HRV-C, which emerged in 2006 and includes 49 designated serotypes [4,5]. The RNA genome of RV, a single-stranded positive-sense RNA, spans about 7.1–7.2 kb. It is structured with 5' and 3'-UTRs, along with a coding region consisting of approximately 2150 codons. This coding region is further divided into the P1 (coding for structural proteins), P2, and P3 (coding for non-structural proteins) regions. The 5'UTR, crucial for viral RNA translation and replication, contains the IRES sequence and a cloverleaf structure. The 3'UTR forms a conserved stem-loop structure preceding a poly(A) tail [6]. Notably, a VPg protein is covalently bound to the 5'-end of the genome.

For this reason, the primers used in this experiment targeted the 5'UTR region, chosen to detect a broad range of serotypes due to its conserved region. The information for the reporter (fluorescence) and quencher for the probe is 5'-HEX (or FAM), 3'-BHQ1, respectively. Supplementary Table 1 provides a comprehensive list of all the assays utilized to generate the figures in this study. References are provided for assays that were either designed "in-house" or obtained from publications.

3. Digital PCR protocol (QX200 Droplet Digital PCR System (Bio-Rad))

Detailed information about the dPCR method is provided in Section 2.2 of the main Materials and Methods, along with specific details outlined in Supplementary Table 2. In summary, reactions were prepared with a final volume of 22.0 μL (10% excess), assembled in a 96-well plate with the relevant assay(s) and compatible supermix, incorporating 5 μL of RNA template.

In the case of singleplex reactions, droplets were categorized using manual thresholds in QuantaSoft v.1.7.4.0917, utilizing the combined wells feature. The threshold position(s) were guided by positive controls and no template control (NTC) reactions, following the guidelines outlined in [7].

4. dPCR assay validation

Validation details for each assay employed in generating the figures within this study are provided in Supplementary Table 3.

5. dPCR data analysis

The copy number concentration was determined using the equation to yield copies per unit volume or expressed as copies per reaction.

$$C = \lambda \times \left(\frac{1}{V_P}\right) \times D \qquad \text{equation (S1)}$$

For the calculation of the sample concentration (C) of target molecules per unit volume, the most direct approach employ, where Vp represents the average volume of a partition, and D denotes the dilution factor of the sample in the dPCR reaction and the mean concentration of target molecules per partition (λ) [8]. This equation establishes the count of target molecules per unit volume of the sample extract measured by the dPCR. Reporting concentration per sample extract is advisable, as it is method-agnostic and facilitates straightforward comparisons

Copies per μL were imported into GraphPad Prism7 for additional analysis and graph generation. The copy number concentrations, initially generated in Excel, were brought into GraphPad Prism7, and the average concentration among replicates along with the relative coefficient of variance were calculated and reported.

6. Predicted precision and relative uncertainty

The considered sources of uncertainty were evaluated for each target gene. During the conducted assessment, the relative standard uncertainty in manual thresholding was determined by calculating the Relative Standard Deviation (RSD) across three distinct threshold settings based on more than ten independent measurements. Additionally, the standard uncertainty arising from variations in partition volume was computed, assuming a uniform rectangular distribution within the reported range of droplet volumes [9–11]. The RSDs were aggregated by taking the positive square root of the summed squared RSDs to derive a combined relative standard uncertainty. The combined standard uncertainty for each target was then used to generate the expanded uncertainty with a coverage factor of $k = 2.2$ (at a 95% confidence level, with a degree of freedom = 11). Graphical data's error bars depict the mean ± standard deviation.

The dynamic range of dPCR was simulated using identical values of k, which were employed in the calculation of copies per reaction through equation (S1).

7. References

1. Dupouey, J.; Ninove, L.; Ferrier, V.; Py, O.; Gazin, C.; Thirion-Perrier, L.; De Lamballerie, X. Molecular Detection of Human Rhinoviruses in Respiratory Samples: A Comparison of Taqman Probe-, SYBR Green I- and BOXTO-Based Real-Time PCR Assays. *Virol. J.* **2014**, *11*, 1–7, doi:10.1186/1743-422X-11-31.
2. Huang, T.; Wang, W.; Bessaud, M.; Ren, P.; Sheng, J.; Yan, H.; Zhang, J.; Lin, X.; Wang, Y.; Delpeyroux, F.; et al. Evidence of Recombination and Genetic Diversity in Human Rhinoviruses in Children with Acute Respiratory Infection. *PLoS One* **2009**, *4*, doi:10.1371/journal.pone.0006355.
3. Lau, S.K.P.; Yip, C.C.Y.; Tsoi, H.W.; Lee, R.A.; So, L.Y.; Lau, Y.L.; Chan, K.H.; Woo, P.C.Y.; Yuen, K.Y. Clinical Features and Complete Genome Characterization of a Distinct Human Rhinovirus (HRV) Genetic Cluster, Probably Representing a Previously Undetected HRV Species, HRV-C, Associated with Acute Respiratory Illness in Children. *J. Clin. Microbiol.* **2007**, *45*, 3655–3664, doi:10.1128/JCM.01254-07.
4. Simmonds, P.; McIntyre, C.; Savolainen-Kopra, C.; Tapparel, C.; Mackay, I.M.; Hovi, T. Proposals for the Classification of Human Rhinovirus Species C into Genotypically Assigned Types. *J. Gen. Virol.* **2010**, *91*, 2409–2419, doi:10.1099/vir.0.023994-0.
5. Palmenberg, A.C.; Spiro, D.; Kuzmickas, R.; Wang, S.; Djikeng, A.; Rathe, J.A.; Fraser-liggett, C.M.; Liggett, S.B. Sequencing and Analyses of All Reveal Structure and Evolution. *Science (80-.).* **2009**, *324*, 55–60.
6. Esneau, C.; Bartlett, N.; Bochkov, Y.A. *Rhinovirus Structure, Replication, and Classification*; Elsevier Inc., 2019; ISBN 9780128164174.
7. Whale, A.S.; Devonshire, A.S.; Karlin-Neumann, G.; Regan, J.; Javier, L.; Cowen, S.; Fernandez-Gonzalez, A.; Jones, G.M.; Redshaw, N.; Beck, J.; et al. International Interlaboratory Digital PCR Study Demonstrating High Reproducibility for the Measurement of a Rare Sequence Variant. *Anal. Chem.* **2017**, *89*, 1724–1733, doi:10.1021/acs.analchem.6b03980.

8. Košir, A.B.; Divieto, C.; Pavšič, J.; Pavarelli, S.; Dobnik, D.; Dreo, T.; Bellotti, R.; Sassi, M.P.; Žel, J. Droplet Volume Variability as a Critical Factor for Accuracy of Absolute Quantification Using Droplet Digital PCR. *Anal. Bioanal. Chem.* **2017**, *409*, 6689–6697, doi:10.1007/s00216-017-0625-y.
9. Pinheiro, L.B.; Coleman, V.A.; Hindson, C.M.; Herrmann, J.; Hindson, B.J.; Bhat, S.; Emslie, K.R. Evaluation of a Droplet Digital Polymerase Chain Reaction Format for DNA Copy Number Quantification. *Anal. Chem.* **2012**, *84*, 1003–1011, doi:10.1021/ac202578x.
10. Corbisier, P.; Pinheiro, L.; Mazoua, S.; Kortekaas, A.M.; Chung, P.Y.J.; Gerganova, T.; Roebben, G.; Emons, H.; Emslie, K. DNA Copy Number Concentration Measured by Digital and Droplet Digital Quantitative PCR Using Certified Reference Materials. *Anal. Bioanal. Chem.* **2015**, *407*, 1831–1840, doi:10.1007/s00216-015-8458-z.
11. Dong, L.; Meng, Y.; Sui, Z.; Wang, J.; Wu, L.; Fu, B. Comparison of Four Digital PCR Platforms for Accurate Quantification of DNA Copy Number of a Certified Plasmid DNA Reference Material. *Sci. Rep.* **2015**, *5*, doi:10.1038/srep13174.

Supplementary Table S1. dPCR oligonucleotide design and target information

Assay ID	Oligo ID	Oligonucleotide sequence and modifications (5′ to 3′)	Design method (software)	In silico verification	Supplier (Catalogue number)	Published Reference
RV	RV_Forward	agcctgcgtggctgcc	Not supplied	Not supplied	Genotech	[1]
	RV_ Reverse	gaaacacggacacccaaagtagt				
	RV_Probe	ctccggcccctgaatgtggctaa				

Supplementary Table S2. dPCR protocol

Component	QX200™ Droplet Digital PCR System (Bio-Rad)
Supermix (µL)	5
Reverse transcriptase (µL)	2
300 mM DTT (µL)	1
Primer-probes mix (µL)<br>(1 µL of 10 µM forward primer, 1 µL of 10 µM reverse primer,<br>1 µL of 5 µM probe)	3
DW (µL)	4
Template (µL)	5
Loaded reaction volume (µL)	20
Reaction partitioning	QX200 droplet generator
Partition reading	QX200 droplet reader
Analysis Software	QuantaSoft v.1.7.4.0917

Supplementary Table S3. dPCR assay validation and data analysis

	Experiment set up									Partition classification method				Assay validation				Description of experimental replication			
**Figure in this study**	**Description of dPCR experimental design**	**dPCR instrument**	**Master mix**	**Assay format and type**	**Assay ID**	**[Final] primer/probe (μM)**		**Other components**	**Annealing temperature (°C)**	**Analysis Software**	**Threshold setting process**	**Classification mode and software tools**	**Details of negative and positive of controls**	**Details of optimisation performed**	**Analytical specificity and LoB**	**Analytical sensitivity/LoD**	**Testing for inhibitors**	**Technical**	**Repeatability and Reproducibility**	**Number of partitions measured (whole exp)**	**Normalisation method**
2	Assay optimization: primer and probe concentration	QX200	One Step RT-ddPCR Advanced Kit for Probes	Singleplex	RV	RV_F<br>RV_R<br>RV_P	0.5 μM<br>0.5 μM<br>0.25 μM	None	60	QuantaSoft v.1.7.4.0917	Manual	One-color plot with manual threshold	Triplicate NTCs, four point dilution series	not performed	No amplification of any targets in buffered solution	not performed	n/a	Triplicate reactions for each dilution	n/a		n/a
3	Assay optimization: primer and probe concentration	QX200	One Step RT-ddPCR Advanced Kit for Probes	Singleplex	RV	RV_F<br>RV_R<br>RV_P	0.5 μM<br>0.5 μM<br>0.25 μM	None	60	QuantaSoft v.1.7.4.0917	Manual	One-color plot with manual threshold	NTCs to match triplicate reactions for each sample	not performed	No amplification of any targets in buffered solution	not performed	n/a	Triplicate reactions	n/a	Ave : 161421.2<br>SD : 17366.3<br>RSD : 10.76	n/a
4A	Assay optimization: primer and probe concentration	QX200	One Step RT-ddPCR Advanced Kit for Probes	Singleplex	RV	RV_F<br>RV_R<br>RV_P	0.5 μM<br>0.5 μM<br>0.25 μM	None	60	QuantaSoft v.1.7.4.0917	Manual	One-color plot with manual threshold	NTCs to match triplicate reactions for each sample	not performed	No amplification of any targets in buffered solution	not performed	n/a	Triplicate reactions	n/a		n/a
4B	Assay optimization: primer and probe concentration	QX200	One Step RT-ddPCR Advanced Kit for Probes	Singleplex	RV	RV_F<br>RV_R<br>RV_P	0.5 μM<br>0.5 μM<br>0.25 μM	None	60	QuantaSoft v.1.7.4.0917	Manual	One-color plot with manual threshold	NTCs to match triplicate reactions for each sample	not performed	No amplification of any targets in buffered solution	not performed	n/a	Triplicate reactions	n/a		n/a
5	Assay optimization: primer and probe concentration	QX200	One Step RT-ddPCR Advanced Kit for Probes	Singleplex	RV	RV_F<br>RV_R<br>RV_P	0.5 μM<br>0.5 μM<br>0.25 μM	None	60	QuantaSoft v.1.7.4.0917	Manual	One-color plot with manual threshold	NTCs to match triplicate reactions for each sample	not performed	No amplification of any targets in buffered solution	not performed	n/a	Triplicate reactions	n/a		n/a

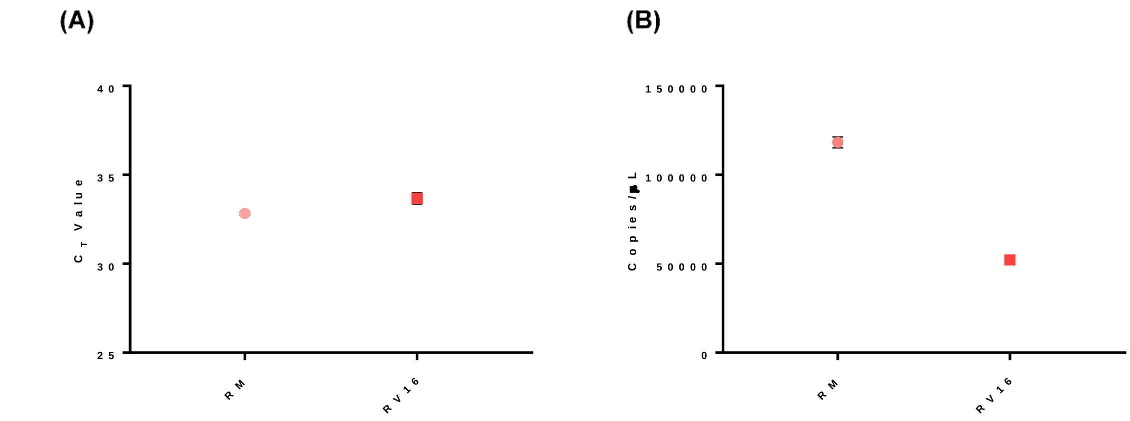

Figure S1. Comparison of human RV RM vs. commercial kit templates. Experimental comparison between human RV RM and commercial control samples using the RV assay for A) qPCR and B) ddPCR.

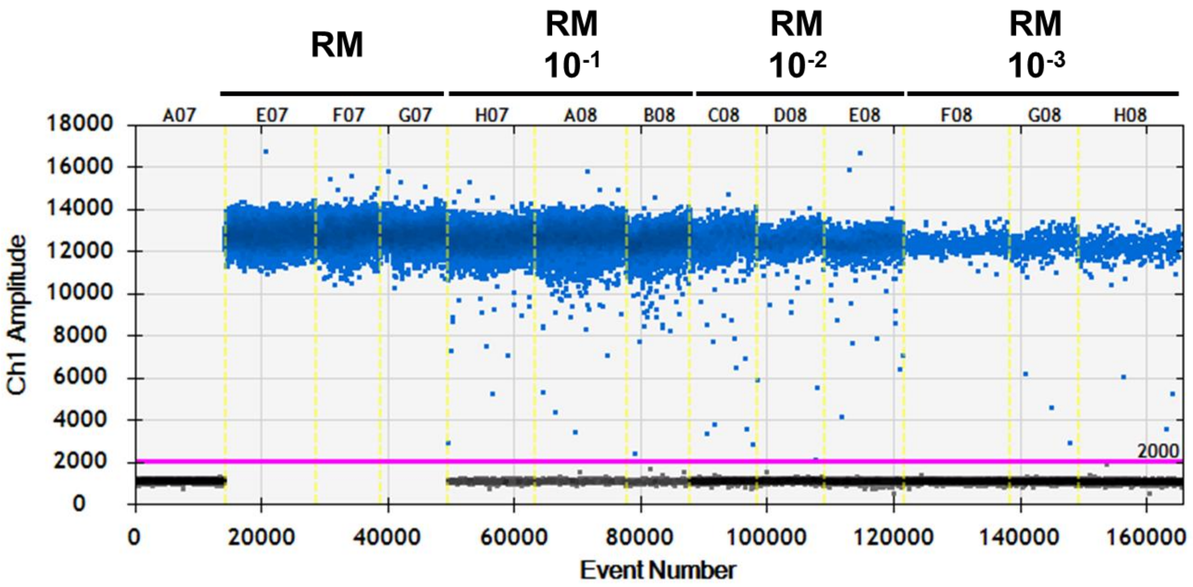

Figure S2. 1D-Amplitude plots of a ddPCR using human RV RM.

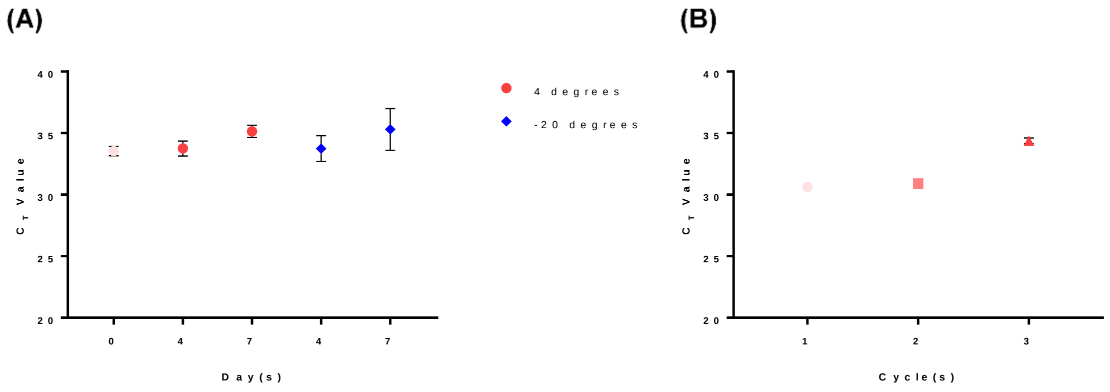

Figure S3. RT-qPCR data for the RM. We conducted qPCR experiments for the previously performed A) Short term stability and B) freeze and thawing. The results indicate a similarity to our previous ddPCR data.
